# Publisher Correction: CRISPR-GPT for agentic automation of gene-editing experiments

**DOI:** 10.1038/s41551-025-01589-0

**Published:** 2025-12-08

**Authors:** Yuanhao Qu, Kaixuan Huang, Ming Yin, Kanghong Zhan, Dyllan Liu, Di Yin, Henry C. Cousins, William A. Johnson, Xiaotong Wang, Mihir Shah, Russ B. Altman, Denny Zhou, Mengdi Wang, Le Cong

**Affiliations:** 1 Department of Pathology, Department of Genetics, Cancer Biology Program, Stanford University School of Medicine, Stanford, CA USA; 2 Center for Statistics and Machine Learning, Department of Electrical and Computer Engineering, Princeton University, Princeton, NJ USA; 3 Department of Computing, Data Science, and Society, University of California, Berkeley, Berkeley, CA USA; 4 Department of Computer Science, University of California, Berkeley, Berkeley, CA USA; 5 Department of Medicine, Stanford University School of Medicine, Stanford, CA USA; 6 Medical Scientist Training Program, Stanford University School of Medicine, Stanford, CA USA; 7 Department of Bioengineering, Department of Genetics, Stanford University, Stanford, CA USA; 8 Google DeepMind, Mountain View, CA USA

**Keywords:** Computational biology and bioinformatics, Computational science, CRISPR-Cas systems, CRISPR-Cas systems

Correction to: *Nature Biomedical Engineering* 10.1038/s41551-025-01463-z, published online 30 July 2025.

In the version of the article initially published, in Fig. 4b and d, the light blue and light purple colours were switched in the keys and have now been corrected as seen in Fig. 1 below. In the Fig. 4f key, "GPT-4o" was light blue and has been corrected to light purple and "CRISPR-GPT" was light purple and has been corrected to green. These errors has been corrected in the HTML and PDF versions of the article.

**Fig. 1:**
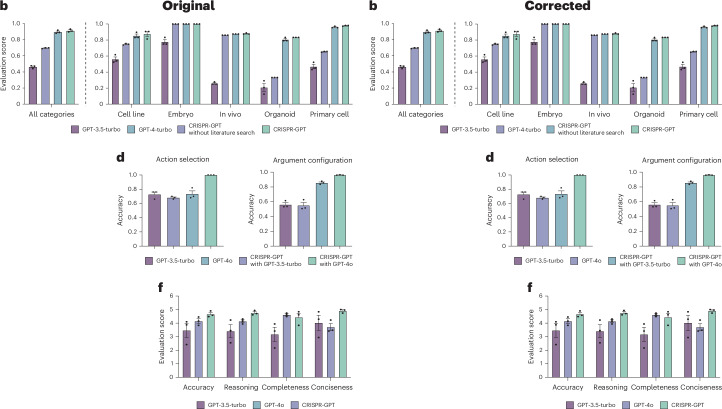
**Original and corrected Fig. 4b, d, f**.

