# Supplementary material for: CRISPR-GPT for agentic automation of gene-editing experiments
Source: Nat Biomed Eng. 2025 Jul 30;10(2):245–58. doi: 10.1038/s41551-025-01463-z (PMC12920143; doi:10.1038/s41551-025-01463-z)
Supplement: Supplementary file 2 — Reporting Summary [file 41551_2025_1463_MOESM2_ESM.pdf]

nature portfolio

Corresponding author(s): Mengdi Wang, Le Cong

Last updated by author(s): May 8, 2025

Reporting Summary

Nature Portfolio wishes to improve the reproducibility of the work that we publish. This form provides structure for consistency and transparency in reporting. For further information on Nature Portfolio policies, see our Editorial Policies and the Editorial Policy Checklist.

Statistics

For all statistical analyses, confirm that the following items are present in the figure legend, table legend, main text, or Methods section.

n/a	Confirmed	
☐	☒	The exact sample size (*n*) for each experimental group/condition, given as a discrete number and unit of measurement
☐	☒	A statement on whether measurements were taken from distinct samples or whether the same sample was measured repeatedly
☐	☒	The statistical test(s) used AND whether they are one- or two-sided<br>*Only common tests should be described solely by name; describe more complex techniques in the Methods section.*
☒	☐	A description of all covariates tested
☒	☐	A description of any assumptions or corrections, such as tests of normality and adjustment for multiple comparisons
☐	☒	A full description of the statistical parameters including central tendency (e.g. means) or other basic estimates (e.g. regression coefficient) AND variation (e.g. standard deviation) or associated estimates of uncertainty (e.g. confidence intervals)
☐	☒	For null hypothesis testing, the test statistic (e.g. *F*, *t*, *r*) with confidence intervals, effect sizes, degrees of freedom and *P* value noted<br>*Give P values as exact values whenever suitable.*
☒	☐	For Bayesian analysis, information on the choice of priors and Markov chain Monte Carlo settings
☒	☐	For hierarchical and complex designs, identification of the appropriate level for tests and full reporting of outcomes
☒	☐	Estimates of effect sizes (e.g. Cohen's *d*, Pearson's *r*), indicating how they were calculated

Our web collection on statistics for biologists contains articles on many of the points above.

Software and code

Policy information about availability of computer code

Data collection	Customized scripts, OpenAI API (gpt-4o-2024-05-13, gpt-3.5-turbo-0125), Primer3 (2.6.1), CRISPResso2 (v2.3.0), CRISPRitz (v2.6.6) were used to within agent. We will deposit a light version of code in github upon publication (https://github.com/cong-lab/crispr-gpt-pub). Full version of code is not freely available, although it has been peer reviewed.
Data analysis	CRISPResso2 (v2.3.0) was used for next-generation sequencing analysis to measure gene-editing efficiencies.

For manuscripts utilizing custom algorithms or software that are central to the research but not yet described in published literature, software must be made available to editors and reviewers. We strongly encourage code deposition in a community repository (e.g. GitHub). See the Nature Portfolio guidelines for submitting code & software for further information.

Data

Policy information about availability of data

All manuscripts must include a data availability statement. This statement should provide the following information, where applicable:

- Accession codes, unique identifiers, or web links for publicly available datasets
- A description of any restrictions on data availability
- For clinical datasets or third party data, please ensure that the statement adheres to our policy

The main data supporting the results in this study are available within the main paper and supplementary information. Additional raw data will be deposit to github (https://github.com/cong-lab/crispr-gpt-pub).

Research involving human participants, their data, or biological material

Policy information about studies with human participants or human data. See also policy information about sex, gender (identity/presentation), and sexual orientation and race, ethnicity and racism.

Reporting on sex and gender	*Use the terms sex (biological attribute) and gender (shaped by social and cultural circumstances) carefully in order to avoid confusing both terms. Indicate if findings apply to only one sex or gender; describe whether sex and gender were considered in study design; whether sex and/or gender was determined based on self-reporting or assigned and methods used. Provide in the source data disaggregated sex and gender data, where this information has been collected, and if consent has been obtained for sharing of individual-level data; provide overall numbers in this Reporting Summary. Please state if this information has not been collected. Report sex- and gender-based analyses where performed, justify reasons for lack of sex- and gender-based analysis.*
Reporting on race, ethnicity, or other socially relevant groupings	*Please specify the socially constructed or socially relevant categorization variable(s) used in your manuscript and explain why they were used. Please note that such variables should not be used as proxies for other socially constructed/relevant variables (for example, race or ethnicity should not be used as a proxy for socioeconomic status). Provide clear definitions of the relevant terms used, how they were provided (by the participants/respondents, the researchers, or third parties), and the method(s) used to classify people into the different categories (e.g. self-report, census or administrative data, social media data, etc.) Please provide details about how you controlled for confounding variables in your analyses.*
Population characteristics	*Describe the covariate-relevant population characteristics of the human research participants (e.g. age, genotypic information, past and current diagnosis and treatment categories). If you filled out the behavioural & social sciences study design questions and have nothing to add here, write "See above."*
Recruitment	*Describe how participants were recruited. Outline any potential self-selection bias or other biases that may be present and how these are likely to impact results.*
Ethics oversight	*Identify the organization(s) that approved the study protocol.*

Note that full information on the approval of the study protocol must also be provided in the manuscript.

Field-specific reporting

Please select the one below that is the best fit for your research. If you are not sure, read the appropriate sections before making your selection.

☒ Life sciences ☐ Behavioural & social sciences ☐ Ecological, evolutionary & environmental sciences

For a reference copy of the document with all sections, see nature.com/documents/nr-reporting-summary-flat.pdf

Life sciences study design

All studies must disclose on these points even when the disclosure is negative.

Sample size	All sample sizes are provided in figure legends.
Data exclusions	No data are excluded.
Replication	Model evaluations are replicated. Number of replication is stated in figure legends. Gene-editing results (next generation sequencing and flow cytometry) do not have replications as the results presented are observational and aim to demonstrate human-AI collaboration allowing beginner researcher to successfully accomplish single gene-editing experiment but not to demonstrate novel biological insights.
Randomization	Models evaluation are on same set of requests. Gene-editing results are on same set of cell lines. No randomization was applied as it's not applicable.
Blinding	Model evaluation are blinded. No blind was applied for gene-editing results as it's not applicable.

Reporting for specific materials, systems and methods

We require information from authors about some types of materials, experimental systems and methods used in many studies. Here, indicate whether each material, system or method listed is relevant to your study. If you are not sure if a list item applies to your research, read the appropriate section before selecting a response.

Materials & experimental systems

n/a	Involved in the study	
☐	☒	Antibodies
☐	☒	Eukaryotic cell lines
☒	☐	Palaeontology and archaeology
☒	☐	Animals and other organisms
☒	☐	Clinical data
☐	☒	Dual use research of concern
☒	☐	Plants

Methods

n/a	Involved in the study	
☒	☐	ChIP-seq
☐	☒	Flow cytometry
☒	☐	MRI-based neuroimaging

Antibodies

Antibodies used	B7-H6 Monoclonal Antibody (JAM1EW), PE (eBioscience™) (1:100 dilution), Anti-CD66a/c/e Mouse Monoclonal Antibody (PE [Phycoerythrin]) [clone: ASL-32] (1:100 dilution)
Validation	All antibodies were well-recognized clones in the field and validated by the manufacturers.

Eukaryotic cell lines

Policy information about cell lines and Sex and Gender in Research

Cell line source(s)	HEK 293T, A549 and A375 cells were obtained from American Type Culture Collection (ATCC).
Authentication	STR analysis was used for cell line authentication.
Mycoplasma contamination	All cells were tested negative for mycoplasma contamination.
Commonly misidentified lines (See ICLAC register)	No commonly misidentified cell lines were used.

Dual use research of concern

Policy information about dual use research of concern

Hazards

Could the accidental, deliberate or reckless misuse of agents or technologies generated in the work, or the application of information presented in the manuscript, pose a threat to:

No	Yes	
☐	☒	Public health
☒	☐	National security
☐	☒	Crops and/or livestock
☐	☒	Ecosystems
☒	☐	Any other significant area

Hazards	CRISPR-GPT agent could automate CRISPR gene-editing experiment design, lower the technical barrier of gene-editing experiments and pose potential misuse of gene-editing technology.

For examples of agents subject to oversight, see the United States Government Policy for Institutional Oversight of Life Sciences Dual Use Research of Concern.

Experiments of concern

Does the work involve any of these experiments of concern:

No	Yes	
☒	☐	Demonstrate how to render a vaccine ineffective
☒	☐	Confer resistance to therapeutically useful antibiotics or antiviral agents
☒	☐	Enhance the virulence of a pathogen or render a nonpathogen virulent
☒	☐	Increase transmissibility of a pathogen
☒	☐	Alter the host range of a pathogen
☒	☐	Enable evasion of diagnostic/detection modalities
☒	☐	Enable the weaponization of a biological agent or toxin
☒	☐	Any other potentially harmful combination of experiments and agents

Precautions and benefits

Biosecurity precautions	Our implementation of CRISPR-GPT agent have 2 layers of biosecurity precautions:<br>Layer 1: keywords filtering during prompt / request. Specifically, for layer 1, a list of keywords are screened<br>Layer 2: addition of explicit warning and consenting step
Biosecurity oversight	Detailed information discussed in the manuscript and supplementary materials.
Benefits	CRISPR-GPT could accelerate the biological discoveries by automating gene-editing experiment design process.
Communication benefits	With the proper precaution, CRISPR-GPT could lead to automated efficient and ethically considerate gene-editing experimental design for biological researchers across different areas and greatly accelerate the biological discoveries.

Plants

Seed stocks	*Report on the source of all seed stocks or other plant material used. If applicable, state the seed stock centre and catalogue number. If plant specimens were collected from the field, describe the collection location, date and sampling procedures.*
Novel plant genotypes	*Describe the methods by which all novel plant genotypes were produced. This includes those generated by transgenic approaches, gene editing, chemical/radiation-based mutagenesis and hybridization. For transgenic lines, describe the transformation method, the number of independent lines analyzed and the generation upon which experiments were performed. For gene-edited lines, describe the editor used, the endogenous sequence targeted for editing, the targeting guide RNA sequence (if applicable) and how the editor was applied.*
Authentication	*Describe any authentication procedures for each seed stock used or novel genotype generated. Describe any experiments used to assess the effect of a mutation and, where applicable, how potential secondary effects (e.g. second site T-DNA insertions, mosiacism, off-target gene editing) were examined.*

Flow Cytometry

Plots

Confirm that:

☒ The axis labels state the marker and fluorochrome used (e.g. CD4-FITC).

☒ The axis scales are clearly visible. Include numbers along axes only for bottom left plot of group (a 'group' is an analysis of identical markers).

☒ All plots are contour plots with outliers or pseudocolor plots.

☒ A numerical value for number of cells or percentage (with statistics) is provided.

Methodology

Sample preparation	A375 cells in culture were detached from the plates, washed twice with PBS, stained with antibodies for 30 mins at 4 degress Celsus and then washed twice with PBS.
Instrument	CytoFLEX (Beckman)
Software	CytExpert Software V2.6, FlowJo V10
Cell population abundance	The purity of the A375 cell line was confirmed by flow cytometry and the purity was above 90%.
Gating strategy	The cells were gated on FSC-A/SSC-A based on the location known to contain A375 cells. Doublets were excluded based on

Gating strategy	FSC-A/FSC-H gating. sgRNAs construct expression was gated on FSC-A/PacBlue-A. dCas9 activation construct was gated on mCherry-A/FITC-A. NCR3LG1 and CEACAM1 expression were gated on PE signal compared to the controls.

☒ Tick this box to confirm that a figure exemplifying the gating strategy is provided in the Supplementary Information.
